# Confined Crystallization of Pigment Red 146 in Emulsion Droplets and Its Mechanism

**DOI:** 10.3390/nano9030379

**Published:** 2019-03-06

**Authors:** Xianze Meng, Yongli Wang, Xin Li, Xue Chen, Dongjun Lv, Chuang Xie, Qiuxiang Yin, Xuling Zhang, Hongxun Hao

**Affiliations:** 1 National Engineering Research Center of Industrial Crystallization Technology, School of Chemical Engineering and Technology, Tianjin University, Tianjin 300072, China; mengxianze@tju.edu.cn (X.M.); xinlll@tju.edu.cn (X.L.); acxie@tju.edu.cn (C.X.); qxyin@tju.edu.cn (Q.Y.); xlzhang_00@tju.edu.cn (X.Z.); 2 Collaborative Innovation Center of Chemical Science and Engineering (Tianjin), Tianjin 300072, China; 3 Shandong YuHong New Pigment Co., Ltd., Dezhou 253000, China; yhchenxue@126.com (X.C.); lvdongjun@163.com (D.L.)

**Keywords:** pigment, PR 146, confined crystallization, miniemulsion, nanoparticle

## Abstract

In this work, the effect of confined space on crystallization processes of pigments was investigated by using C.I. Pigment Red 146 (PR 146) as a model compound. The colloidal system (i.e., emulsion droplets) was used as a nanoreactor to prepare nanoscale PR 146 for the inkjet printer. The effects of the space confinement were investigated by comparing the products of PR 146 prepared from bulk solution, macroemulsion, and miniemulsion. The results showed that PR 146 crystallized in mini-emulsion had the narrowest particle size distribution and the average particle size can be as small as 172.5 nm, one order of magnitude smaller than the one obtained from the bulk solution. X-ray diffraction (XRD) data revealed that PR 146 crystallized in all three solutions where the crystalline state and had similar crystallite sizes. The process mechanism of crystallization confined in the miniemulsion droplets was proposed and explained. The function mechanism of the co-stabilizer during the crystallization of PR 146 in emulsion was also explained. It was found that sodium chloride could counteract the pressure difference as an osmotic pressure agent and prevent the migrating of water from small droplets into big droplets. The influences of dosages of emulsifiers and co-stabilizers on droplet size and the size of the obtained PR 146 particles were evaluated and the optimal conditions were determined. Furthermore, the disparity of PR 146 products prepared by different methods was investigated by UV–Vis spectra. The aqueous dispersion of PR 146 crystallized in miniemulsion had the highest absorbance and darkest color.

## 1. Introduction

In the field of textile printing, in the last few decades, increasing attention has been devoted to inkjet printing systems [1,2]. Aside from the small manufacturing scale and customized production, inkjet printing technology is attractive because of its low pollution, quick response, rational ink management, and so on [3,4,5,6]. As for the inks, pigment-based inks have been widely used due to their good washing and light fastness [7]. However, most pigments are insoluble in the inks and tend to agglomerate [8], and hence are likely to clog the nozzles of the inkjet printer (around 1 μm) [9]. In this regard, a number of approaches have been used to prepare nano-pigments with good dispersion, such as polymer coating, organic-inorganic hybridization, and dispersant additives [10,11].

In recent years, using colloidal systems for the crystallization processes to prepare nanomaterials has become increasingly popular. The application of emulsion droplets as templates is one of the ways. The emulsion droplets can be considered as nanoreactors and confine the spaces where crystallization occurs, thereby controlling the size and shape of materials [12]. This method has been used for preparing highly specific surface area catalysts [13,14] and hollow spheres of metals and oxides [15,16,17]. Emulsions typically include macroemulsions, miniemulsions, and microemulsions, which are distinguished by sizes of emulsion droplets. Macroemulsions require only a small amount of emulsifier and have a particle size of more than 1 micron, which are unstable systems. Additional co-stabilizers are needed for the miniemulsions that have a particle size of about 200 nm and are kinetically stable systems. Microemulsions need a high concentration of emulsifier and they are thermodynamically stable systems with particle sizes below 100 nm [18]. From the perspective of cost, miniemulsions are more suitable for large-scale production of nanomaterials because of its small particle size and low emulsifier consumption. To date, reports about confined crystallization of pigments in miniemulsion systems were rarely reported.

**Figure 1:**
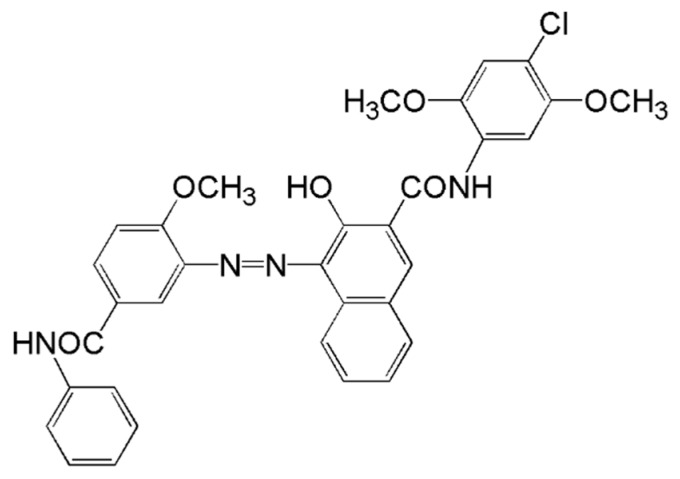
Chemical structure of C.I. pigment red 146.

In this work, the effects of space confinement on the crystallization of pigments were investigated by using C.I. pigment red 146 (abbreviated as PR 146 in this work) as the model compound. C.I. pigment red 146 whose structure is shown in Figure 1 is an azo pigment widely used for textile dyeing. Three different crystallization ways including crystallization in bulk solution, crystallization in macroemulsion and crystallization in miniemulsion were used to prepare PR 146. The latter two methods were carried out within the droplets of water-in-oil emulsions because the reactants are easily soluble in water. The emulsifier span 80 was employed to form the emulsion system and NaCl was used as a co-stabilizer for the miniemulsion due to its strong hydrophilia. The particle size, crystallinity, morphology, and absorbance of different products were characterized to explore the effect of the space confinement on products. Influences of the emulsifier and co-stabilizer content were also investigated by comparing droplet size and PR 146 particle sizes. In addition, the dispersibility of PR 146 products crystallized in bulk solution, macroemulsion, and miniemulsion was investigated by UV–Vis spectra.

## 2. Materials and Methods

### 2.1. Materials

3-Amino-4-methoxybenzanilide and 4-Chloro-3-hydroxy-2,5-dimethoxy-2-naphthanilide (naphthol AS-LC) were provided by Yuhong Pigment Co., Ltd., Shandong, China. Sodium hydroxide (NaOH, 96%) and hydrochloric acid (HCl, 36–38%) were purchased from Tianjin Sixth Chemical Reagent Factory, Tianjin, China. Cyclohexane (99%), sodium nitrite (NaNO_2_, 99%), and sodium chloride (NaCl, 99%) were purchased from Yuanli Chemical Co., Ltd., Tianjin, China. Sorbitan monooleate (Span 80, 95%) was purchased from Kermel Chemical Co., Ltd., Tianjin, China. All the reactants and solvents were used as received. Deionized water was used for all the experiments.

### 2.2. Crystallization of PR 146 in Bulk Solution

First, NaOH (0.50 g) was dissolved in 50 mL water and naphthol AS-LC (3.17 g) was added into the obtained solution. The mixture was heated up and kept at 85 °C for 30 min under magnetic stirring (300 rpm) to obtain clear solution. Next, 3-Amino-4-methoxybenzanilide (2.08 g) was dissolved in 65 mL HCl (0.4 M), and the solution was cooled down to 0 °C. NaNO_2_ (0.60 g) was added to the solution with gentle shaking to obtain a yellow solution. Finally, the former solution was dropwise added into the latter one within 10–15 min under magnetic stirring (200 rpm). After keeping the reaction for another 1 h, the mixture was filtered, washed with water, and dispersed in water.

### 2.3. Crystallization of PR 146 in Macroemulsion

First, 0.06 g Naphthol AS-LC was added into 4 g cyclohexane. After ultrasonication for 10 min, Naphthol AS-LC was uniformly dispersed in the organic phase. Next, 0.04 g 3-Amino-4-methoxybenzanilide was dissolved into 1.3 mL HCl (0.4 M), and the obtained solution was mixed with 5.2 g cyclohexane. Certain amount of Span 80 (1 wt %, 5 wt % or 10 wt %) was added, and the mixture was sonicated for 20 min. Finally, these two mixtures were mixed together and 0.02 g NaNO_2_ was added simultaneously to trigger the reaction. After reacting under magnetic stirring (300 rpm) for 1 h, the suspension was centrifuged (10,000 rpm, 5 min) to collect the product. Then, the obtained solid product was washed once with ethanol, once with water, and dispersed in water.

To make sure that (*Z*)-4-methoxy-3-(methyldiazenyl)-*N*-phenylbenzamide (abbreviated as KD in this work, one reactant, see Section 3.2) will not diffuse into the continuous phase, a model experiment consisting of surfactant-free two-phase system was carried out. 0.2 g 3-Amino-4-methoxybenzanilide was dissolved into 6.5 mL HCl (0.4 M), and the solution was mixed with 26 g cyclohexane. The mixture was sonicated for 20 min and then allowed to rest for 2 min. Thereafter, the KD distribution was evaluated by the homogeneity of color.

### 2.4. Crystallization of PR 146 in Miniemulsion

First, Naphthol AS-LC was dispersed in the organic phase (see Section 2.3). Next, 0.04 g 3-Amino-4-methoxybenzanilide and 1.5 wt % NaCl (0.10 g) were dissolved into 1.3 mL HCl (0.4 M), and the solution was mixed with 5.2 g cyclohexane. Certain amount of Span 80 (1 wt %, 3 wt % or 5 wt %) was added, and the mixture was pre-emulsified by stirring for 10 min at 500 rpm. The pre-emulsion was sonicated for 10 min to form a stable miniemulsion. Finally, the former organic phase containing Naphthol AS-LC was dropwisely added to the miniemulsion under magnetic stirring (300 rpm) and 0.02 g NaNO_2_ was added simultaneously and slowly to trigger the reaction. Subsequent processing is the same as in the case of macroemulsion.

### 2.5. Characterization Methods

The particle size distributions of emulsion and PR 146 were analyzed by using dynamic light scattering (Malvern Zetasizer Nano ZS, Malvern Instruments Ltd., Worcestershire, UK). The instrument was equipped with a He-Ne laser lamp (0.4 mW) with wavelength of 633 nm. Measurements were performed at 25 °C in insulated chamber using dynamic light scattering technique.

X-ray diffraction (XRD) was performed with a D/max-2500 (Rigaku, Tokyo, Japan) diffractometer (Cu Kα radiation, λKα1 = 1.5406 Å) over diffraction angle (2θ) from 2° to 50° with a scanning rate of 0.067° s^−1^ at 100 mA and 40 kV.

Scanning electron microscopy (SEM) was performed in a field emission scanning electron microscope (Gemini SEM 300, ZEISS, Oberkochen, Germany) with an extractor voltage of 10.0 kV. Samples for SEM characterization were prepared by drop-casting diluted dispersions on carbon conductive tape and spraying gold.

The UV-Vis spectra of PR 146 aqueous dispersions were obtained with a spectrophotometer (UV-2600, SHIMADZU, Kyoto, Japan).

## 3. Results and Discussion

### 3.1. Analysis of the Water Droplets in Emulsions

In order to study the effect of space limitation on product crystallization, water droplets in emulsions were first analyzed. In this work, cyclohexane containing Span 80 was used as the continuous phase. Water was used as the dispersed phase. Span 80 was selected as a nonionic emulsifier due to its low hydrophilic−lipophilic balance (HLB = 4.3), which tends to form inverse emulsion [19]. Ultrasound was employed to generate shear forces to promote the emulsification of the two phases. To resist Ostwald ripening, NaCl was used as co-stabilizer in the water phase of the miniemulsion because of its strong hydrophilia.

**Figure 2:**
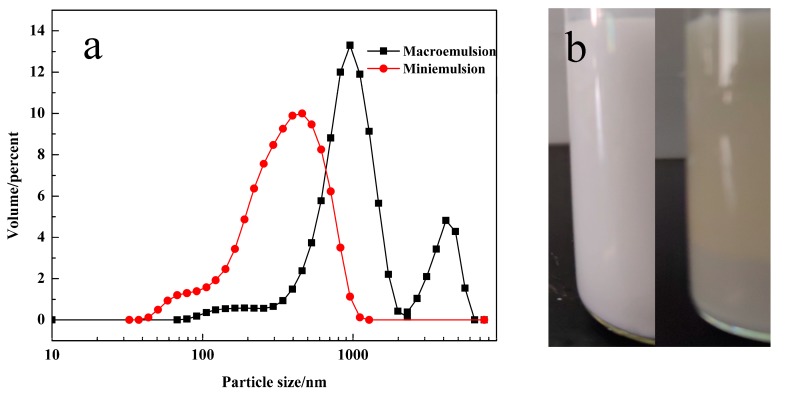
(**a**) Particle size distributions of macroemulsion and miniemulsion. (**b**) Photographs of macroemulsion just after ultrasound and after 3 h of rest (left and right, respectively).

The droplet size distributions of macroemulsion and miniemulsion were measured immediately after emulsification (unless mentioned otherwise, the mass fraction of emulsifier for the macroemulsion and miniemulsion was 1 wt % and 3 wt %, respectively). The results are shown in Figure 2a. The average particle sizes of these two emulsions were 612.3 nm and 251.8 nm, respectively. Further, the PDI’s (Polymer dispersity index) were 0.507 and 0.195, respectively, suggesting that droplets in miniemulsion had a narrower particle size distribution. Besides, the two emulsions differed greatly in stability, as shown in Figure 2b. The macroemulsion was obviously stratified in 3 h, while the miniemulsion could remain un-stratified for more than 3 days.

### 3.2. Determination of the Reaction Conditions in Emulsions

**Scheme 1:**
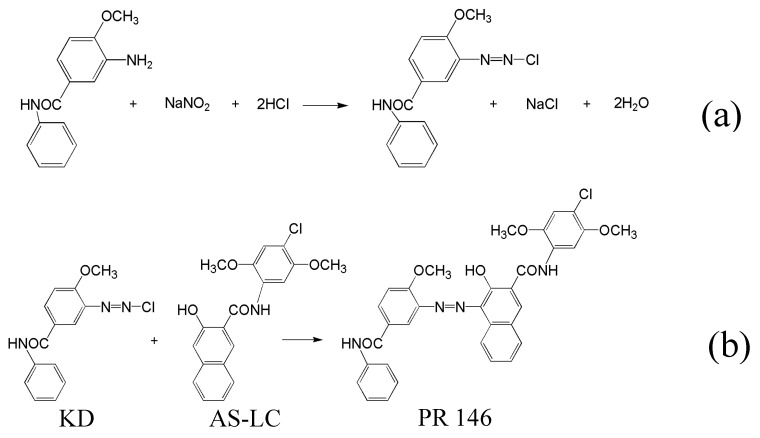
Synthesis process of PR 146: (**a**) 3-Amino-4-methoxybenzanilide diazotization; (**b**) coupling reaction of KD and naphthol AS-LC.

The synthesis of PR 146 red 146 is divided into two steps. A diazonium salt KD is formed by adding sodium nitrite to hydrochloric acid containing 3-Amino-4-methoxybenzanilide (Scheme 1a). Then, PR 146 is synthesized in water by coupling the diazonium salt KD with naphthol AS-LC (Scheme 1b).

**Figure 3:**
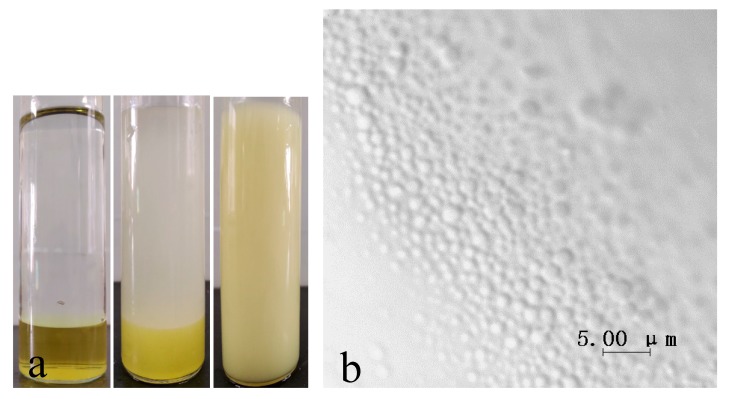
(**a**) Photographs of two-phase system. The left is before mixing, the middle is without emulsifier, the right is with emulsifier. (**b**) Microphotograph of water droplets in macroemulsion.

To make sure that the reaction will happen in water droplets, the reactant KD should be confined to the dispersion phase. Therefore, a model experiment consisting of surfactant-free two-phase system was carried out to verify that KD will not diffuse into the continuous phase. As shown in Figure 3a, the water phase containing KD is yellow before mixing. For the surfactant-free two-phase system, after half an hour of ultrasound and 3 min of rest, the oil phase was almost colorless, indicating that KD was kept in the water phase. Additionally, the color of the macroemulsion is uniform because water droplets were evenly dispersed in the organic phase. The same conclusion can be drawn from the microscopic photograph of macroemulsion containing reactant KD (Figure 3b). KD is insoluble in cyclohexane and no crystals can be found in the continuous phase. In the meantime, it can be seen from the photograph that diameters of most droplets in the macroemulsion were between 500–2000 nm, which is consistent with the results of dynamic light scattering.

**Figure 4:**
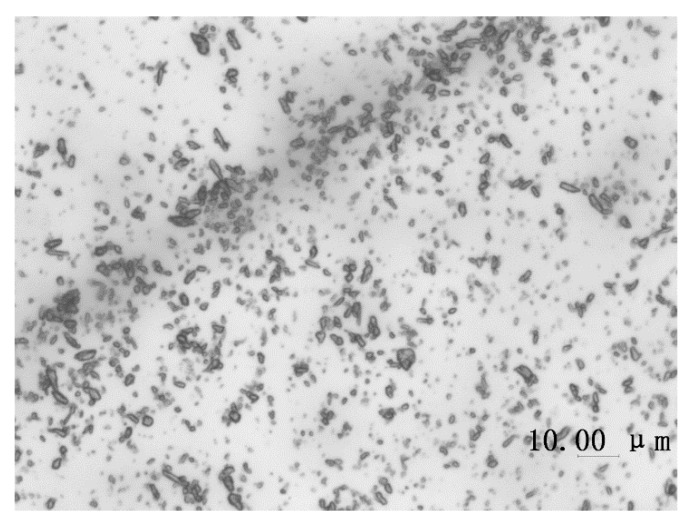
Microphotographs of naphthol AS-LC dispersed in cyclohexane.

Another reactant Naphthol AS-LC is also insoluble in cyclohexane. It was dispersed in the continuous phase before the reaction. The results of ultrasonic dispersion of naphthol AS-LC in cyclohexane are shown in Figure 4. The particles were evenly distributed, which is conducive to diffuse into water droplets under stirring.

### 3.3. Confined Crystallization within Emulsion Droplets

**Figure 5:**
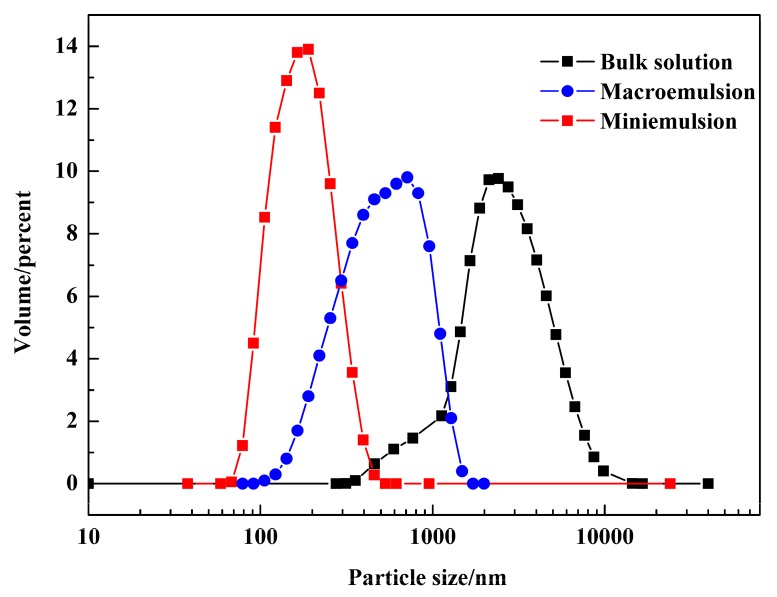
Particle size distributions of PR 146 obtained from the bulk solution, macroemulsion, and miniemulsion.

To explore the effect of space confinement on product crystallization, PR 146 products were prepared from three different crystallization methods: The bulk solution crystallization, macroemulsion crystallization, and miniemulsion crystallization. The particle sizes of the obtained PR 146 by different methods were analyzed, and the results are shown in Figure 5. It can be found that the particle size distribution of PR 146 obtained from miniemulsion was the smallest while most uniform, and all particles were below 500 nm. Most particles of PR 146 obtained from macroemulsion were in the range of 300–1000 nm. Additionally, PR 146 obtained from the bulk solution had the biggest particles with size ranging between 500 and 6000 nm. It could be found that the size distribution of PR 146 was related to the crystallization space. PR 146 products, obtained from the miniemulsion, can meet the size demand of inkjet ink.

**Figure 6:**
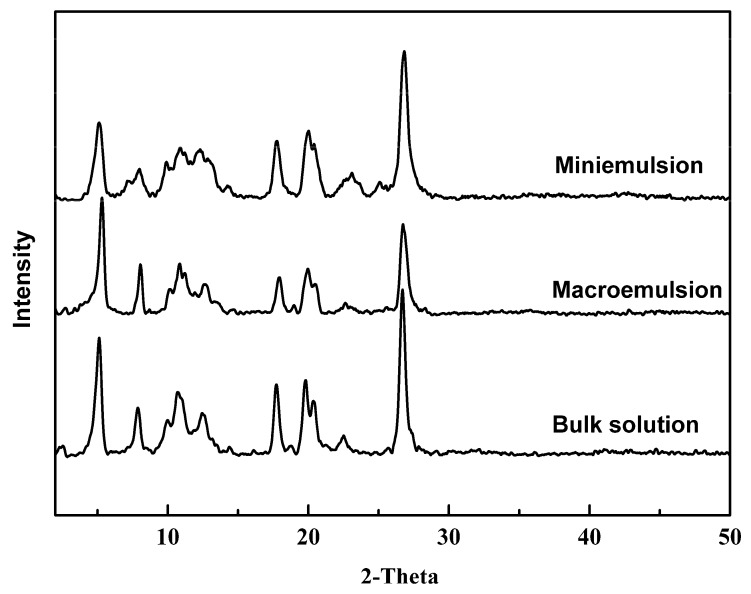
X-ray diffractograms of PR 146 obtained from the bulk solution, macroemulsion, and miniemulsion.

X-ray diffraction (XRD) data can provide information not only about crystal form and crystallinity of the crystalline materials, but also about their crystallite size. The diffractograms of pigments produced from bulk solution, macroemulsion, and miniemulsion are shown in Figure 6. It can be found that all the products have the same XRD patterns, indicating that all products crystallized from different methods were the same crystal form. Furthermore, the crystallinity of the products obtained from macroemulsion or miniemulsion is nearly the same with the crystallinity of product from bulk solution. This is one advantage of macroemulsion or miniemulsion over microemulsion, which tends to form an amorphous product for its minuscule space.

The values of crystallite size, determined by the Scherrer formula [20], are shown in Table 1. It can be found that crystallite sizes of the products obtained by the three ways differed little. By comparing crystallite sizes and particle sizes determined by dynamic light scattering, it can be concluded that the particles crystallized from bulk solution were composed of hundreds of crystallites, whereas the size of the particles crystallized from macroemulsion and miniemulsion were comparable with ten to twenty times the crystallite size.

**Figure 7:**
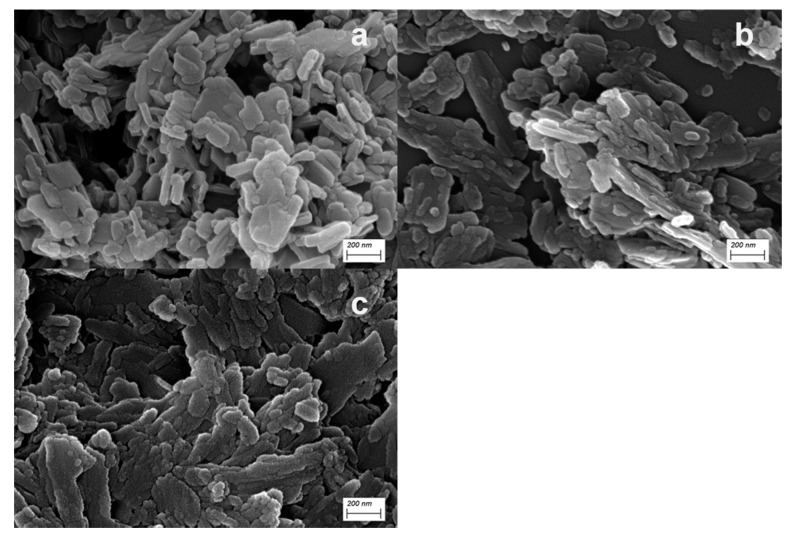
SEM micrographs of PR 146 obtained from: (**a**) miniemulsion, (**b**) macroemulsion, and (**c**) bulk solution.

The morphology and particle size of PR 146 prepared from different solutions were also analyzed by SEM, and the results are shown in Figure 7. The morphology of the three products were all flaky or rod-like. Compared with the other two products, PR 146 products obtained from miniemulsion were smaller in size and more uniform in size distribution. Additionally, PR 146 products obtained from bulk solution obviously had a larger size.

### 3.4. Process Mechanism for Crystallization in Confined Space

**Scheme 2:**
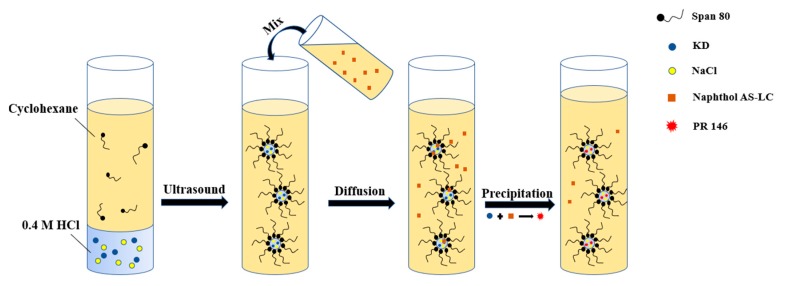
Schematic description of the crystallization mechanism of PR 146 confined in miniemulsion droplets.

The processes mechanism of PR 146 crystallization confined in miniemulsion is described in Scheme 2. Inverse emulsion was formed under the function of ultrasound. The water droplets were covered by surfactants to prevent them from coalescing. The reactant KD and co-stabilizer NaCl were dissolved and confined to water droplets. Then, the organic phase containing naphthol was added. Because of its high solubility in water, the reactant naphthol AS-LC can cross the liquid-liquid interface and dissolve into the water droplets. With the diffusion of naphthol, the precipitation could occur at the liquid–liquid interface or inside the droplets. Nucleation and growth of the crystal were confined by the small space, thus forming nano-scale PR 146. The separation of the product was accomplished by subsequent centrifugation steps. The crystallization processes in the macroemulsion were similar with that shown in Scheme 2, except that no co-stabilizer was used.

**Scheme 3:**
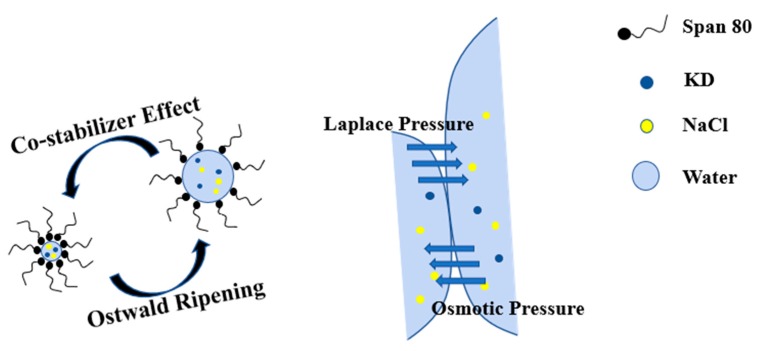
Schematic description of function mechanism of co-stabilizer.

In the process of emulsification, the high shear forces can generate small water droplets in the presence of emulsifier. However, the Laplace pressure (i.e., the pressure difference between the inner and outer part of a curved surface caused by the surface tension at the liquid-liquid interface) is higher for small droplets than for bigger ones, which results in the disappearance of small droplets (i.e., Ostwald ripening). The immediate cause is that water molecules diffuse across the interface from small droplets to larger ones. As a result, the droplet size distribution of the macroemulsion eventually widens and becomes larger. In our work, the additional sodium chloride was used for the formation of the miniemulsion to resist Ostwald ripening. The function mechanism of co-stabilizer is described in Scheme 3. For the miniemulsion, the hydrophilic substance (i.e., sodium chloride) can counteract the pressure difference as an osmotic pressure agent and prevents the migrating of water from small droplets into big droplets [21]. When two water droplets come into contact, the interface is equivalent to a semipermeable membrane due to the presence of a surfactant. Water molecules diffuse from small to large droplets under the Laplace pressure described above. This increases the concentration of sodium chloride in the smaller droplets, allowing water molecules to diffuse from the larger droplets to the smaller ones under osmotic pressure. The two effects are balanced to keep the droplet size at the nanometer scale. These nanometer scale spaces act as nanoreactors and allow the control of both the nucleation and growth of products due to the confinement space of the crystallization process.

### 3.5. Effect of Emulsifier and Co-Stabilizer Dosage

**Figure 8:**
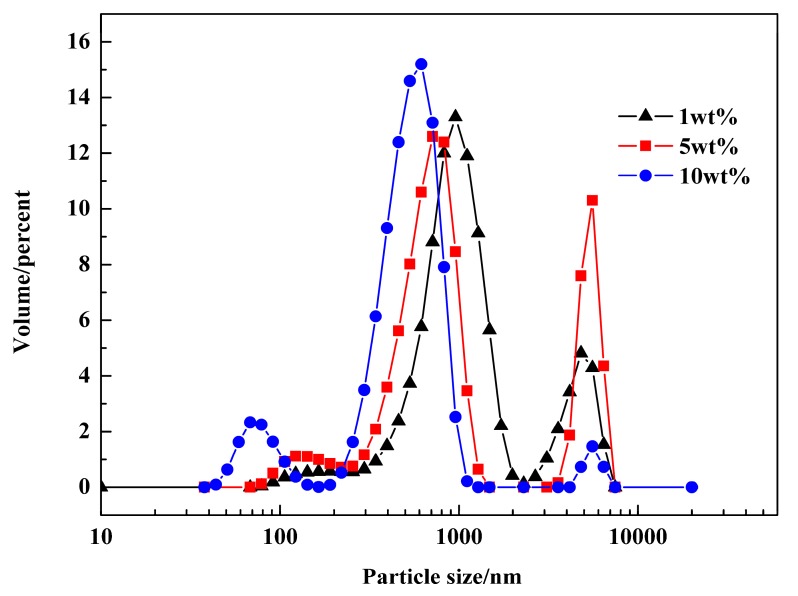
Size distributions of macroemulsions containing different dosages of emulsifier.

The sizes and distributions of droplets in macroemulsions containing different dosages of emulsifier (i.e., Span 80) were analyzed, and the results are shown in Figure 8. The amounts of Span 80 in the three groups were 1 wt %, 5 wt % and 10 wt %, respectively. It could be found that size distributions of these three macroemulsions were similar and inhomogeneous. They all have some droplets of 4–6 micron in size, caused by the Ostwald ripening. The average sizes were 612.3 nm, 711.4 nm, and 595.4 nm, respectively. The difference of average particle size was mainly caused by inhomogeneous size distribution. It seems that the dosage of emulsifier had little effect on the particle size distribution of macroemulsion. It should also be noticed that all three groups of macroemulsions were unstable during the experiments.

**Figure 9:**
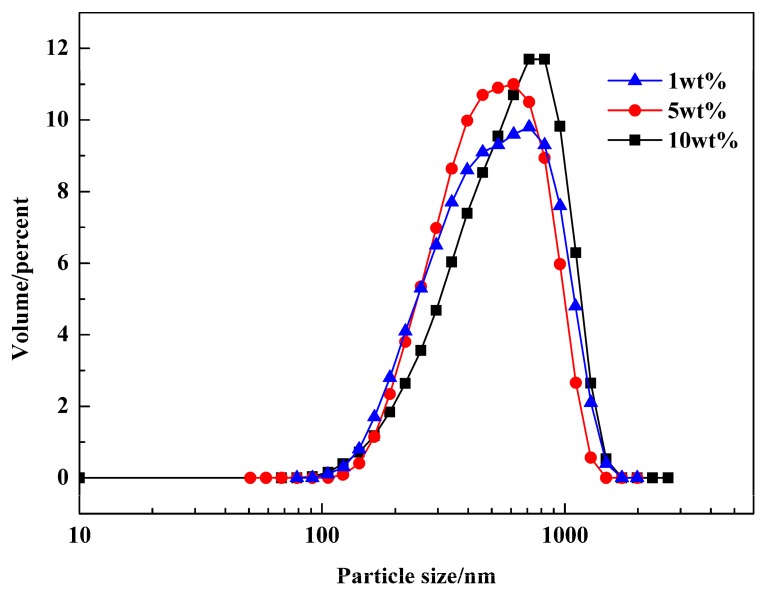
Particle size distributions of PR 146 products obtained from macroemulsions containing different dosages of emulsifier.

The size distributions of PR 146 products prepared from macroemulsions containing 1 wt %, 5 wt % and 10 wt % Span 80 were also analyzed, and the results are shown in Figure 9. It could be found that there is no significant difference in particle size distribution between the three experiments, and the average particle sizes of the obtained products were 378.2 nm, 361.4 nm, and 384.5 nm, respectively. Since the amount of emulsifier in the range of 1–10 wt % had little effect on the water droplet size of macroemulsion, it is reasonable that it had little effect on the size of the obtained particles.

**Figure 10:**
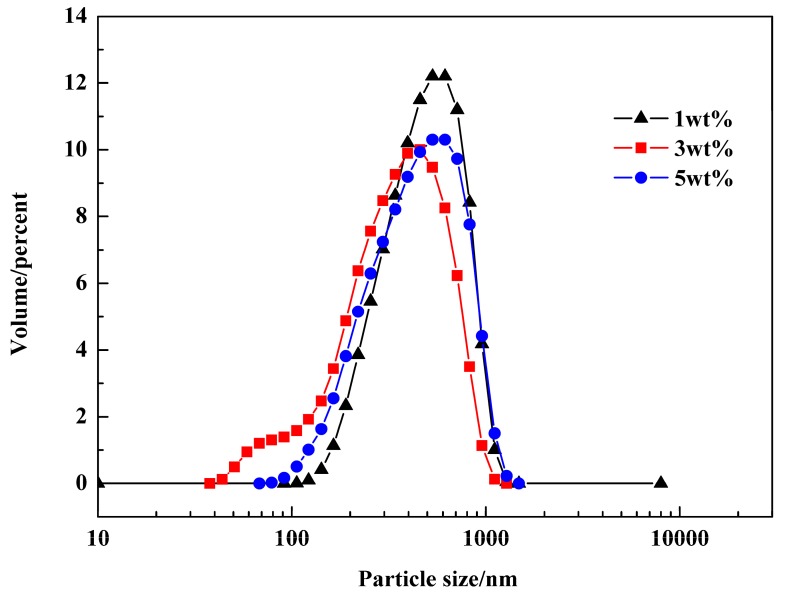
Size distributions of miniemulsions containing different dosages of emulsifier.

Similarly, the size distributions of droplets in miniemulsions containing different dosages of emulsifier were analyzed, and the results are shown in Figure 10. The amounts of Span 80 in the three groups were 1 wt %, 3 wt %, and 5 wt %, respectively. The amount of co-stabilizer (i.e., NaCl) were all 1.5 wt %. It could be found that the size distributions of the three miniemulsions had little difference, although the size of the droplets from the system containing 3 wt % emulsifier was smaller. The average sizes of different droplets were 299.8 nm, 251.8 nm, and 271.4 nm, respectively.

**Figure 11:**
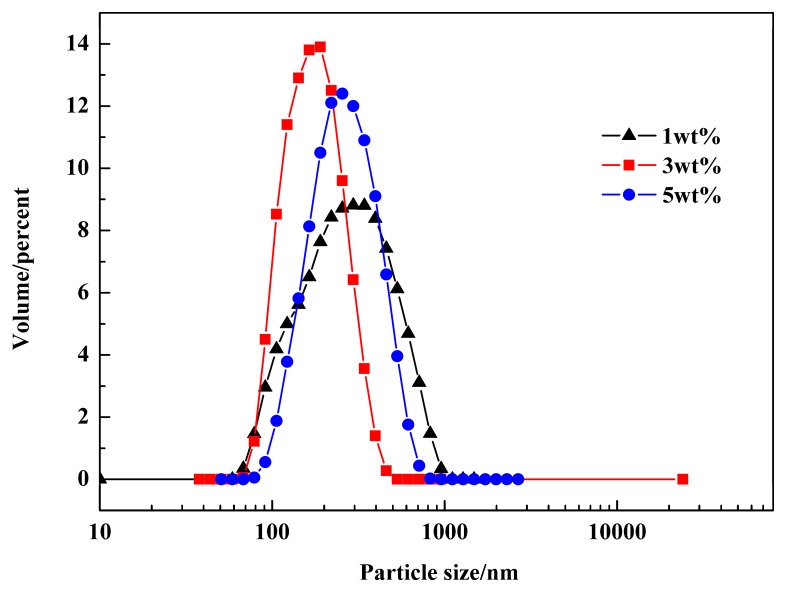
Particle size distributions of PR 146 obtained from miniemulsions containing different dosages of emulsifier.

Additionally, the particle size distributions of PR 146 products prepared from miniemulsions containing different dosages of emulsifier were analyzed, and the results are shown in Figure 11. The amount of emulsifier and co-stabilizer was the same as that in the droplets experiment. It could be found that the particle size distribution of pigment prepared with 3 wt % Span 80 was uniform, with most particle sizes ranging from 70 nm to 400 nm, and the average particle size was 172.5 nm. However, when the dosage of Span 80 changed into 1 wt % or 5 wt %, the average particle size increased by about 100 nm, and the number of large particles with a size over 300 nm was also increased obviously. Compared with the conclusion of miniemulsion droplet size, it can be inferred that the small change of miniemulsion droplet size can significantly affect the product size. The results indicated that the emulsifying effect was the best when the dosage of emulsifier was 3 wt %, and the pigment with the best particle size distribution can be obtained.

**Figure 12:**
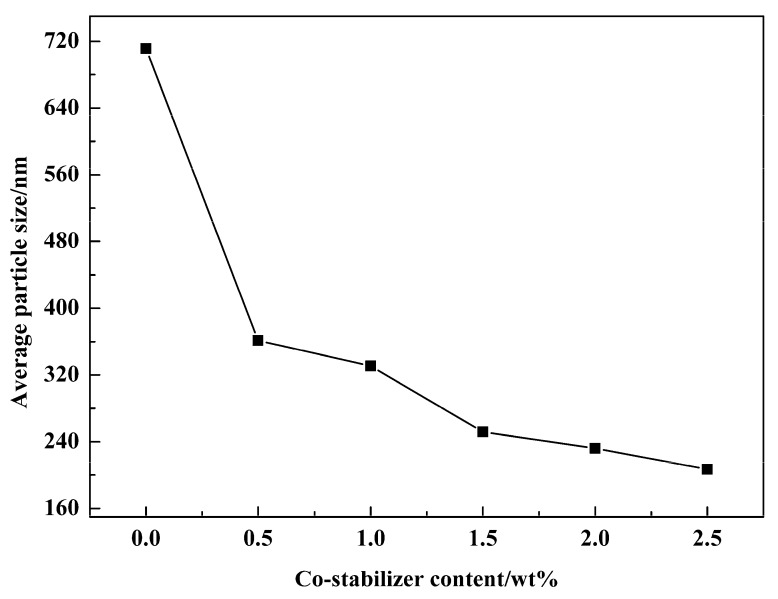
Average particle size of miniemulsions obtained by using a different amount of co-stabilizer.

According to the formation mechanism of miniemulsion, the amount of co-stabilizer could also influence the size of droplets of miniemulsion and hence influence the properties of the final product. Therefore, the effect of different doses of co-stabilizer on the droplets in miniemulsion was also investigated. In all experiments, the amount of emulsifier was set constantly at 3 wt %. The results are shown in Figure 12. It could be found that the co-stabilizer effect was significant. A content of only 0.5 wt % could reduce the droplet’s average particle size below 400nm, and the droplet’s particle size was further decreased with the increasing of the content. It could be inferred from the formation mechanism that increased co-stabilizer content will result in higher osmotic pressure under the same conditions, which will counteract Laplace pressure between large and small droplets and therefore prevent droplets from becoming larger.

**Figure 13:**
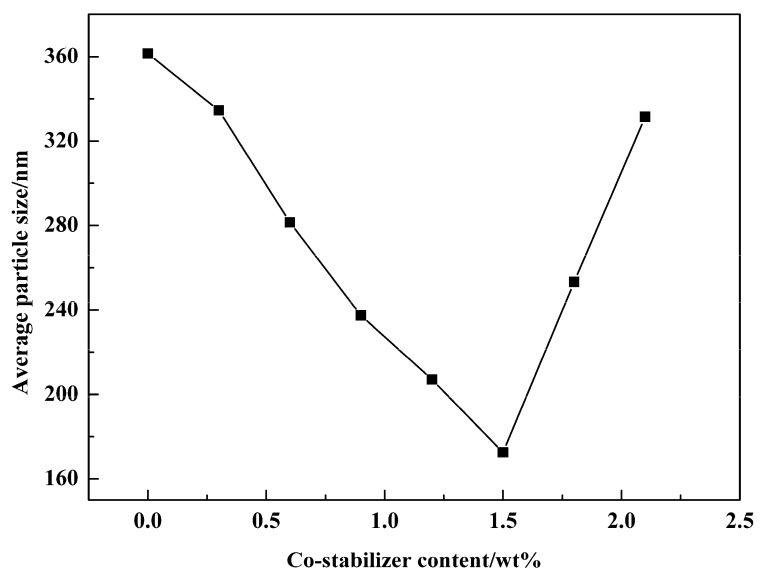
Average particle size of PR 146 obtained from miniemulsions with different co-stabilizer content.

Next, the effect of different doses of co-stabilizer on the product was also investigated, and the results are shown in Figure 13. With the increasing of the amount of co-stabilizer, average particle size of products decreased firstly and reached the minimum value as the amount of co-stabilizer was 1.5 wt %. Then, average particle size increased significantly after the amount of co-stabilizer exceeded 1.5 wt %. Co-stabilizer NaCl can stabilize the water droplets by counteracting the pressure difference and allows the crystallization process to occur in nanoscale space. When the amount of NaCl was increased, the water droplets became smaller and more stable, which will help to suppress the growth of crystals. However, when the amount of NaCl was too high, the solubility of KD in water decreased, and part of the reactant precipitated. In the mixing process, part of the reaction will then occur in the continuous phase, and the space confinement effect will be weakened, resulting in larger particle size.

### 3.6. UV-Vis Spectra of the Samples

**Figure 14:**
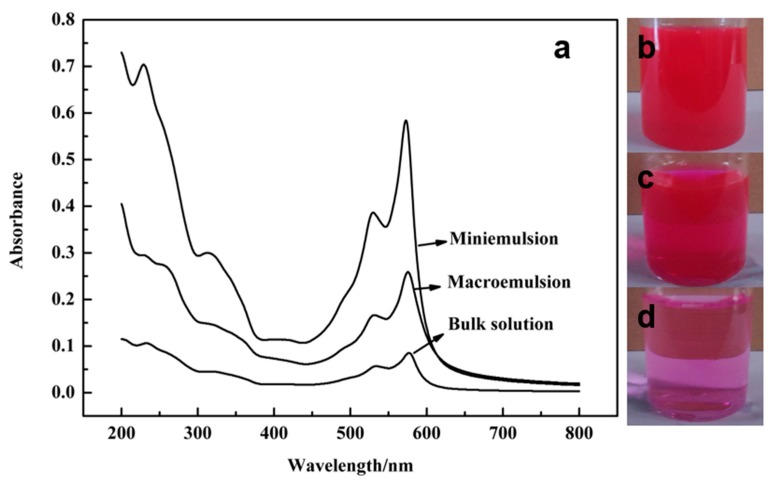
UV-Vis absorption spectra (**a**) and photographs of PR 146 aqueous dispersions prepared from the miniemulsion (**b**), macroemulsion (**c**), and bulk solution (**d**).

To evaluate the effect of confined crystallization on the performance of the products, the absorbance of PR 146 products prepared from three different solutions was analyzed by UV-Vis spectra. Aqueous dispersions of PR 146 products prepared by three different methods were prepared. The solid content was controlled at 0.000396% by dilution so that their absorbance can be less than 0.8, which can ensure the accuracy of the results. The results are shown in Figure 14. It could be seen that the aqueous dispersion of PR 146 crystallized from the miniemulsion showed a much higher absorbance in the region of 200–600 nm, indicating better dispersion [21]. The absorbance of the aqueous dispersion of pigment crystallized from the bulk solution was the lowest. This is consistent with the results of the above particle size analysis. From the photographs, it could be seen that pigment aqueous dispersions of PR 146 prepared from miniemulsion showed darker colors. The solid content was verified by drying and weighing after the end of the experiment.

## 4. Conclusions

The confined crystallization of C.I. pigment red 146 within water droplets of water-in-oil macroemulsion and miniemulsion demonstrated that space confinement will affect the crystal nucleation and growth, and hence will affect the particle size. Under the confinement of “nanoreactors” provided by miniemulsion droplets, the average particle size of PR 146 could be as small as 172.5 nm and the particle size distribution was much more uniform, meeting the size requirements of inkjet printer. XRD data indicated that the PR 146 crystallized from bulk solution, macroemulsion and miniemulsion are the same crystalline form. The mechanism of the confined crystallization processes in miniemulsion droplets and the role of co-stabilizer in resisting Ostwald ripening were proposed and explained. The effects of different doses of emulsifier and co-stabilizer on the emulsion droplets and precipitated PR 146 products were investigated. Additionally, the optimum dosage of emulsifier and co-stabilizer was determined by a series of experiments. The UV-Vis spectra analysis results showed that PR 146 products obtained from miniemulsion exhibited the highest absorbance and better dispersity in water.

## Figures and Tables

**Table 1:** Average particle size measured by dynamic light scattering and crystallite size calculated by applying the Scherrer formula.

Crystallization Method	Average Particle Size/nm	Crystallite Size/nm
Bulk solution	2840.0	17.2
Macroemulsion	361.4	19.6
Miniemulsion	172.5	15.8
